# Design and Shape Optimization of Strain Gauge Load Cell for Axial Force Measurement for Test Benches

**DOI:** 10.3390/s22197508

**Published:** 2022-10-03

**Authors:** Omar Sabah Al-Dahiree, Mohammad Osman Tokhi, Nabil Hassan Hadi, Nassar Rasheid Hmoad, Raja Ariffin Raja Ghazilla, Hwa Jen Yap, Emad Abdullah Albaadani

**Affiliations:** 1 Department of Mechanical Engineering, College of Engineering, University of Baghdad, Baghdad 10071, Iraq; 2 Department of Mechanical Engineering, Faculty of Engineering, University of Malaya, Kuala Lumpur 50603, Malaysia; 3 School of Engineering, London South Bank University, London SE1 0AA, UK; 4 Department of Aeronautical Engineering, College of Engineering, University of Baghdad, Baghdad 10071, Iraq; 5 Department of Electrical Engineering, Faculty of Engineering, University of Malaya, Kuala Lumpur 50603, Malaysia

**Keywords:** strain gauge, load cell, machine design, axial force measurement, shape optimization, finite element method (FEM), Wheatstone bridge, amplifier circuit

## Abstract

The load cell is an indispensable component of many engineering machinery and industrial automation for measuring and sensing force and torque. This paper describes the design and analysis of the strain gauge load cell, from the conceptional design stage to shape optimization (based on the finite element method (FEM) technique) and calibration, providing ample load capacity with low-cost material (aluminum 6061) and highly accurate force measurement. The amplifier circuit of the half Wheatstone bridge configuration with two strain gauges was implemented experimentally with an actual load cell prototype. The calibration test was conducted to evaluate the load cell characteristics and derive the governing equation for sensing the unknown load depending on the measured output voltage. The measured sensitivity of the load cell is approximately 15 mV/N and 446.8 µV/V at a maximum applied load of 30 kg. The findings are supported by FEM results and experiments with an acceptable percentage of errors, which revealed an overall error of 6% in the worst situation. Therefore, the proposed load cell meets the design considerations for axial force measurement for the laboratory test bench, which has a light weight of 20 g and a maximum axial force capacity of 300 N with good sensor characteristics.

## 1. Introduction

For decades, experimental solid mechanics and, more broadly, industry and engineering applications have faced a critical need for reliable and high-throughput measurement of forces and moments [1]. Load cells, commonly referred to as force transducers, are considered accurate force-measuring devices. They have a range of industrial uses in addition to scientific and technological research and development [2]. Load cells have been providing quality measurements in robotics and automation [3,4,5], agriculture [6,7], medicine [8,9,10], industrial weighing [11,12], and many other applications for decades [13,14,15,16,17,18,19,20,21,22]. The load cell can also be used to gauge the interference force in wearable robotic applications (such as exoskeletons and prostheses) or to detect the comparison force as the robot walks to provide information to the stable-controlling system [23,24,25,26,27]. Due to the wide range of potential applications, load cells are crucial.

Physical force testing prototypes and products are essential in product development and research. Axial force testing is used, for example, in the validation and approval of products in the product development process and the parameterization and verification of models and simulations. It also generates knowledge and findings in product development and research [28]. Load cells were utilized to determine the axial force integrated with the test bench for laboratory measurement. Many examples can be found on axial force test benches based on the load cell, such as the syringe test stand, catheter track force test, wire bond testing, spring testing systems, medical valve sensors (TAVR fatigue testing), and prosthetic hip fatigue testing. In addition, thrust (axial) force measurements can be found in rocket propellant systems and underwater vehicles [29,30]. For example, the work in [29] suggests new tension and compression test tools that prevent fluctuation of the measured force of specimen deformation during hardening behaviors for sheet metal forming and spring-back in the automotive industry. The main idea behind the novel device is to attach the load cell between the specimen mount and the test device’s fixed jig regardless of jig vibration. The load cell inside the novel test apparatus can therefore be used to quantify the axial force of a specimen during deformation.

In another work [30], a low-cost physical test bench is modified to identify the thruster parameters for underwater vehicle applications. To prevent the need to seal the sensor, the load cell is placed out of the water on the test bench, which comprises a water tank and a thruster. On Wheatstone bridge measurement and amplifier equipment, a strain gauge load cell is employed in a test bench to determine the thrust force component.

During vehicle durability tests on the testing ground, significant design revisions should be conducted on the vehicle or engine body when utilizing the standard multi-axial load cell for the road load identification system of the engine mount. Engine mounts connect the internal combustion engines on the vehicle structure. For a feasible solution without any adjustment to vehicle design, a custom multi-axial load cell has been designed for a BCar I4 engine to measure forces and moments on the engine mount. The measured data can be used further for fatigue life estimation [31].

The slotted flanges combined with load cells are designed and validated experimentally to measure a V-band clamp’s axial and radial force distribution, using the Abaqus software’s finite element analysis (FEA) for the design and analysis [32]. In addition, a novel micro-fabricated resonant load cell with an external displacement actuator has been designed and integrated into a hybrid micro-mechanical test bench for the mechanical characterization of small-scale models [33]. Another study [34] presented and experimentally tested different designs of load cells for mini tensile stress measurement for a smaller specimen to obtain the mechanical characteristic.

Under various loading circumstances and varying environmental conditions, the axial force is measured with load cells. Presently, strain test-centered load cells are used to measure most axial forces. These load cells have two ends, one of which can be attached to a solid, sturdy structure and the other carrying the load [35]. The load cells based on strain measures have extremely sensitive gauging capabilities. A proportion of the total capacity can be used to calculate the load cell’s precision. The use of strain measures is essential for obtaining an output that is proportionate to the applied force. These strain measures are fastened to related elastic body parts [35].

The load cell’s performance is principally determined by the dimensions and shape of its elastic composition [36]. Several requirements, including mass, strain, and stress, must be met while creating a load cell [35]. Numerous specialists and academics [1,2,35,36,37,38,39,40] have proposed numerous logical designs for the elastic structures of force sensors and produced positive practical outcomes [36]. Nevertheless, the majority of the weighing load sector is searching for load cells that are compact, robust, and environmentally resistant. These days, new and sophisticated approaches are being developed to accomplish this, which is made possible by combining a numerical simulation method with an optimization strategy. The current product will be altered as a result. There is a huge rise in computer and numerical simulation when finite element analysis is used [35]. Hence, our work has introduced more robust and successful computer simulation-based procedures.

Typically, researchers and engineers intend to purchase a commercial load cell device and its completed amplifier circuit with DAQ signal for their laboratory test bench without prior knowledge of designing, manufacturing, and testing a customized strain gauge load for their particular application. Then, they attempt to install the load cell with adjustments to meet their specific application requirements in size, shape, dimension, and force range capacity. Moreover, it is unreasonable to be obliged to consider the features and properties of commercial load cells when designing particular laboratory test benches. Instead, the force test benches can be designed based on desirable features and properties integrated with custom load cells. However, it is relatively rare in the literature on the design, customization, shape optimization, instrumentation, calibration, and experimental testing of load cells to find them together in one work. The improvement in load cells designed to have an elastic structure could meet application measurement requirements during the consideration and optimization of the elastic body.

This paper’s main aim was to facilize customization of strain gauge load cell based on the desired characteristic during the axial force tests on the proving ground. The proposed study of the load cell can assist in eliminating undesired design and specification revisions for the specimen or test bench and provide excellent sensor characteristics for the load cell. Consequently, it is vital to present the feasibility of designing and customizing the force load cell according to the criteria for static performance. This work introduces the design and analysis of strain gauge load cells that provide medium axial load capacity, low cost, and small size with high-accuracy measurement for laboratory test benches. First, the overall concept is proposed for the load cell configuration, and the amplifier instrumentation and strain gauge are illustrated. Then, the mathematical model and design considerations for the proposed load cell are clarified. The stress–strain analysis based on the FEM technique was conducted to find the optimal dimensions for the load cell’s shape for a given rating load that maximizes the sensor sensitivity. The strain gauge load cell was fabricated with two embedded strain gauges. Following this, the proposed load cell was characterized experimentally. The calibration was carried out by applying a known load and measuring the resulting voltage to derive the load cell’s governing equation and validate the usefulness of the proposed customized load cell for the laboratory test bench.

## 2. Design Concept and Electronic System of the Load Cell

### 2.1. Prototype Configuration

**Figure 1:**
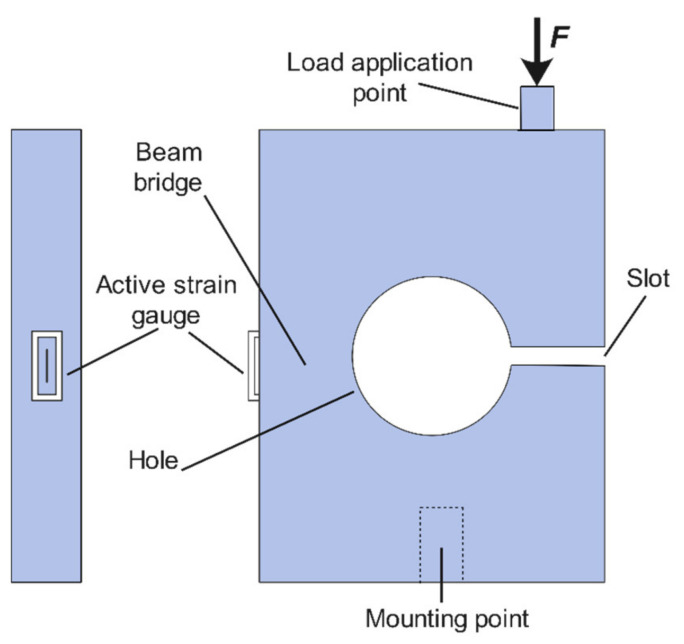
Structure of the load cell.

The load cell structure in this study was optimized and designed based on the conceptual structure [41], as demonstrated in Figure 1. The load cell structure comprises a metal block with a single hole, a small slot, two strain gauges, a load application point, and a mounting point. The metal body is drilled with a single hole in the center and a small slot on the side to generate bending strain on the beam bridge. As shown in Figure 1, the active strain gauge is fixed on the surface side of the load cell body where the beam bridge is located. The axial force is applied and concentrated on the load application point at the thrust (perpendicular) axis. The bottom part of the load cell body is mounted to the ground through one bolt at the mounting point.

### 2.2. Working Principles

A load cell is employed in an electromechanical tool (transducer) to transform a force into an electrical signal. This indirect conversion procedure is divided into two steps. The applied force can be sensed by deforming the strain gauge with a suitable mechanism structure. A strain gauge is a small electrical device showing resistance changes when strained. Then, the strain gauge converts the slight change in resistance into electrical signals using an electrical circuit known as a Wheatstone bridge. The output voltage from the bridge circuit is typically millivolts, which needs increment by a device amplifier.

The overall working concept of the proposed load cell is similar to that of the bending beam type. The applied force on the load cell body mainly causes bending stress on the beam bridge with a linear relationship, as presented in Figure 1. The beam bridge is the main structural element of the load cell, along with the slot. This element is designed to develop a strain directly proportional to the applied load. On the other hand, the load cell structure plays an essential role in generating desirable deflection related to the applied load. This slot attempts to close slightly as the whole body bends a minimal amount. Ultimately, the strain corresponds linearly to the stress of the bridge beam by the load cell material’s elastic modulus (E). The strain gauge is attached to the surface of the load cell body nearest to the bridge beam for accurate strain measurement.

### 2.3. Wheatstone Bridge Circuit

**Figure 2:**
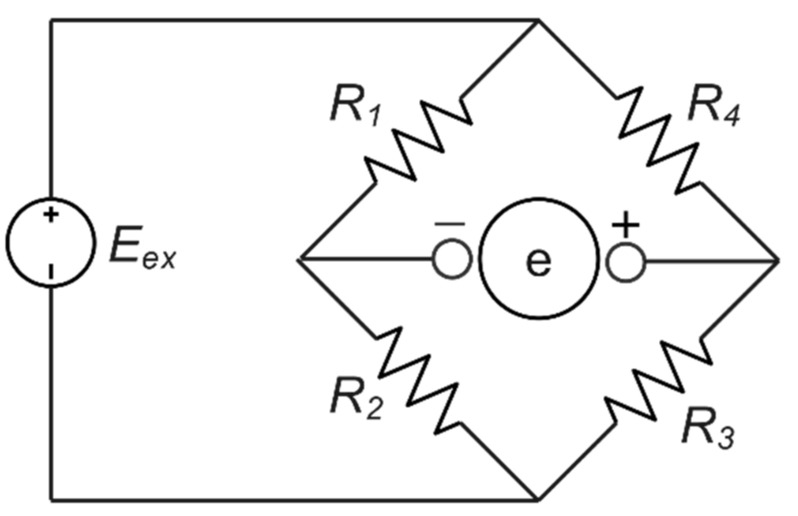
Wheatstone bridge circuit.

The simplest electrical bridge circuit that can measure resistance, inductance, and capacitance is the Wheatstone bridge. A Wheatstone bridge structure can be considered two ballast circuits coupled so that the initial stable-state voltages cancel out when the output voltage e is measured ($R_{1},R_{2}$, and $R_{3},R_{4}$). Figure 2 shows the general Wheatstone bridge, which has four resistive arms and an excitation voltage, $E_{ex}$, applied across it [42]. The output voltage of the bridge, e, will be equivalent to [42]:

(1) $$e=\left[\frac{R_{3}}{\left(R_{3}+R_{4}\right)}-\frac{R_{2}}{\left(R_{1}+R_{2}\right)}\right]\;E_{ex}$$

This equation shows that when $\;R_{1}/R_{2}=R_{4}/R_{3}$, the voltage output e will be zero. A balancing bridge is known as such a configuration in these conditions. However, any minor alterations in resistance in any arm of the bridge can result in a non-zero output voltage [39]. 

With a voltage or current excitation source, strain measures are constantly employed in a bridge configuration. The strain scales are coupled in a Wheatstone bridge configuration to transform the tiny change in resistance into a functional electrical signal. Quarter-bridge, half-bridge, and full-bridge are the three different strain gauge configurations. The type of bridge configuration with a strain gauge in a Wheatstone bridge depends on how many active component legs are present [39].

The gauge factor is a quantitative expression of the strain gauge’s essential property known as sensitivity to strain (GF). The gauge factor is described as the proportion of the fractional transformation in length (strain) to the fractional shift in electrical resistance [42]:

(2) $$GF=\frac{\Delta R/R_{g}}{\Delta L/L}=\frac{\Delta R/R_{g}}{\varepsilon }$$

Any variation in the strain gauge resistance will reduce the bridge’s stability and result in an output voltage that is not zero. If the minimal resistance of the strain gauge is defined as $R_{g}$, then the strain-generated variation in resistance, Δ*R*, can be stated as [42]:

(3) $$\Delta R=\varepsilon *R_{g}*GF$$

**Figure 3:**
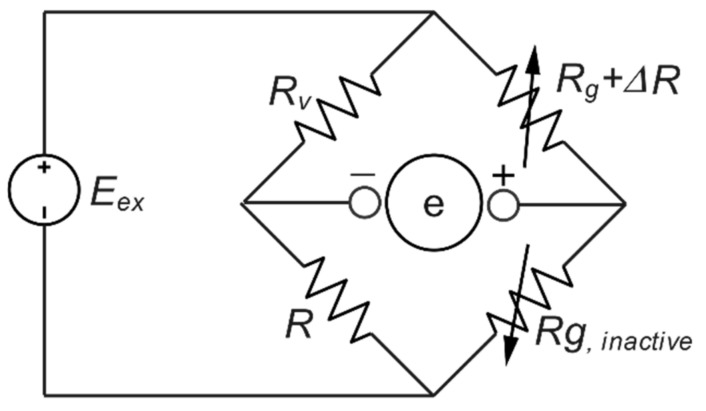
Half-Wheatstone bridge configuration.

**Figure 4:**
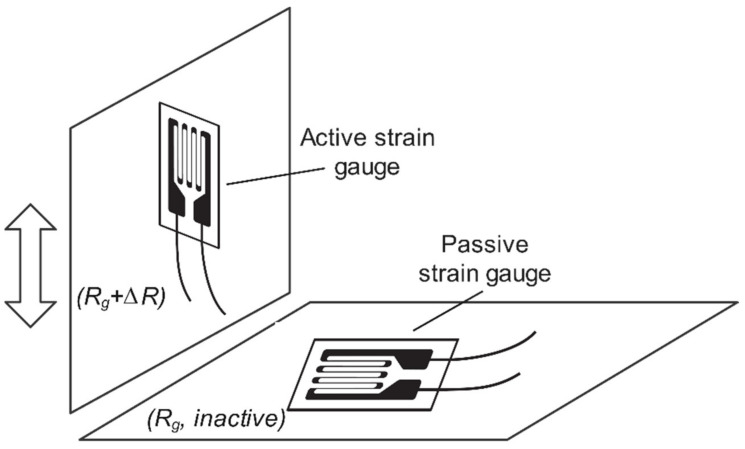
Configuration placement of active and passive strain gauges.

The proposed load cell in this study adopted a half-bridge system with a single active strain gauge component and one passive strain gauge (dummy gauge), a temperature-sensing bridge element (dummy gauge), as shown in Figure 3. The strain gauge configuration is displayed in Figure 4, where the active gauge ($R_{g}$ + Δ*R*) is placed on the bridge beam of the load cell. In contrast, the passive gauge (dummy) is located on the mounting surface to sense the environmental temperature. The effects of temperature variation are minimized, and the circuit will experience slight temperature-encouraged measurement error because both strain gauges will either increase or reduce resistance by a similar percentage in response to alterations in temperature (the voltage and the resistance ratio do not change) [43].

### 2.4. Amplifier Circuit

**Figure 5:**
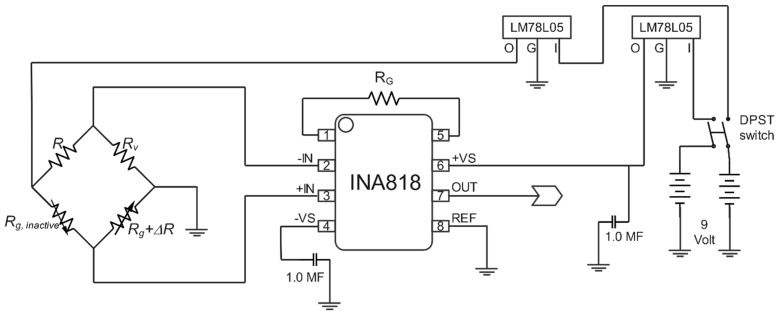
Strain gauge amplifier circuit.

The amplifier circuit works to amplify the output electrical signal of the Wheatstone bridge (a few millivolts) to provide a measurable output voltage with accurate data. Figure 5 reveals an electrical diagram of the amplifier circuit with the Wheatstone bridge that is used in integration with the load cell. As can be seen, the active strain gauge ($R_{g}+\Delta R$), passive (dummy) strain gauge ($R_{g}$*, inactive*), one variable resistor ($R_{v}$*,* potentiometer), and fixed resistor $\left(R\right)$ are organized in a Wheatstone bridge structure. In this, the variable resistor works to balance the bridge and enhance the accuracy of the measurements.

According to Figure 5, the bridge’s output voltage is linked to the amplifier chip’s +IN and -IN terminals, and a digital multimeter is used to measure the output voltage of the amplifier circuit (OUT and REF). The circuit employs two 9 V batteries for the power deliveries and 5 V voltage regulators to supply the voltage inputs to the amplifier chip. A single DPST switch controls the circuit’s voltage. Finally, two capacitors are connected to the positive and negative voltage supplied to the amplifier chip.

The amplifier chip of INA818 (high-precision instrumentation amplifier, Texas Instruments Incorporated, Dallas, TX, USA) is used in the amplifier circuit as it is a known high-precision differential amplifier for low input voltage with low power consumption and runs over a broad array of single supplies. The INA818 provides a high common-mode rejection ratio. Due to its super-beta input transistors, the INA818 offers remarkably low input offset voltage, offset voltage drift, input bias current, and current noise. The INA818 can attain 1 to 10,000 times via an external gain resistor $\left(R_{G}\right)$ producing small signal amplification [44]. Therefore, in this paper, the output signal was amplified (G) by 2001 times, and the gain resistance $\left(R_{G}\right)$ was computed based on the datasheet of INA818, as illustrated in the next section.

#### Selection of Gain Resistance $\left(R_{G}\right)$

The voltage amplification factor (amplifier gain, G) is determined based on the value of the gain resistor ($R_{G})$ of the amplifier chip. The appropriate amplifier gain (G) value depends mainly on the load cell specification design. The relation between the amplifier gain G and fixed gain resistor $R_{G}$ can be adopted from the INA818 Amplifier datasheet and can be expressed as [44]:

(4) $$\;\;R_{G}=\frac{50\;k\Omega }{G-1}$$

Amplifier gain (G) can be measured by [44]:

(5) $$\;\;G=\frac{V_{o}}{e}$$

where $V_{o}$ is the preferred output voltage of the amplifier (4.7 V) and e is the output voltage of the Wheatstone bridge circuit (inputted to the amplifier). The value of e can be calculated by [42]:

(6) $$\;\;e=\frac{GF}{4}\left(\varepsilon_{1}-\varepsilon_{2}+\varepsilon_{3}-\varepsilon_{4}\right)\;E_{ex}$$

where $\varepsilon_{1},\varepsilon_{2},\;\varepsilon_{3},\;$ and $\varepsilon_{4}\;$ are the values of the strain of each gauge, and $E_{ex}$ is the excitation voltage source.

Since the proposed load cell has only one active gauge $\;\varepsilon_{1}=\varepsilon$, the strains $\varepsilon_{2}$,$\;\varepsilon_{3}$, and $\varepsilon_{4}$ are always zero. Then, the value of e can be expressed as:

(7) $$e=\frac{GF}{4}\;\left(\varepsilon \right)*E_{ex}$$

where ε can be obtained from the FEM strain results or theoretical strain of the load cell, and the gauge factor (GF) is a constant value extracted from the used strain gauge datasheet [45]. 

In addition, the strain of the load cell ε can be expressed directly to the output voltage $V_{O}$ by the equation:

(8) $$\varepsilon =\frac{4*V_{O}}{GF*E_{ex}*G}$$

## 3. Shape Design of the Load Cell

### 3.1. Mathematical Model of the Load Cell Unit

**Figure 6:**
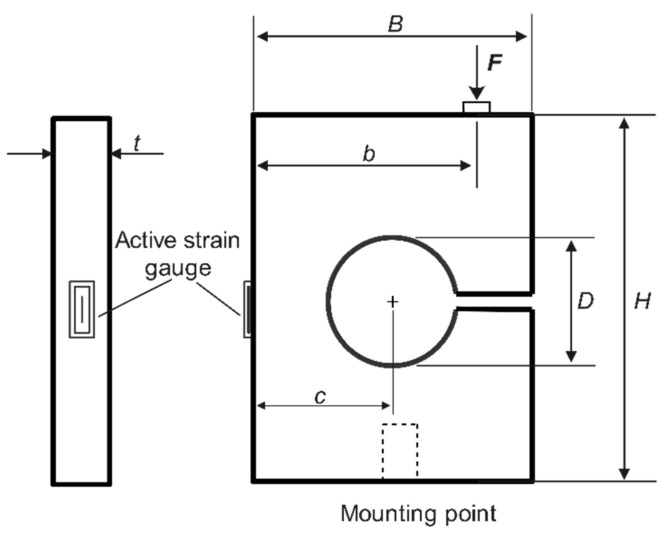
Geometry of the load cell structure.

The proposed load cell is subjected to combined bending and axial stresses, and these stresses are located on the bridge beam part where the strain gauge is mounted. However, the bending stress effect is the dominant component and has a linear relationship with the applied force. Figure 6 schematically shows the geometry parameters of the load cell structure. The load cell is primarily subjected to bending when the external load is applied. Therefore, it is modeled as a beam whose bottom end is fixed to a rigid base, and load is applied on the free side. The applied force is evenly distributed throughout the whole body. The equation describing the maximum bending stress of the load cell can be written by [41]:

(9) $$\sigma_{max}=\frac{4\;F\;\left[\frac{3\;b}{2\;\left(c-\frac{D}{2}\right)}-1\right]}{t\;\left(c-\frac{D}{2}\right)}$$

where *F* is the applied force to the load cell, *t* is the thickness of the load cell, *D* is the hole diameter, *c* is the hole location, and *b* is the location of the applied force.

The maximum strain is related to the load cell’s applied force and material property in Young’s modulus (E). It can be expressed as [41]:

(10) $$\varepsilon_{max}=\frac{4\;F\;\left[\frac{3\;b}{2\;\left(c-\frac{D}{2}\right)}-1\right]}{E\;t\;\left(c-\frac{D}{2}\right)}$$

The strain is inversely correlated with the body thickness (*t*) and the elastic modulus (E) and is linearly proportionate to the exerted load.

### 3.2. Design Considerations

The load cell was developed for initial laboratory bench tests for measuring the axial force. The load cell measured thrust force with a maximum applied torque of 300 Nm (nearly 30 kg). The proposed load cell’s basic dimensions are 30 mm × 30 mm (*B × H*). Other geometrical parameters were subjected to some limitations during the design process of the load cell, as follows [41]:

(11) $$\frac{B}{3}\leq D\leq \frac{2B}{3}$$

(12) $$\frac{B}{8}\leq t\leq \frac{B}{2}$$

(13) $$b\leq \frac{B}{2}$$

Each of these variables has a distinct impact on the load cell’s flexibility, load capacity, and overall strength. Hence, optimization of a parameter based on the FEM experimental design methodology is possible. Table 1 summarizes the design parameters, illustrating the minimum and maximum values. The FEM process was carried out to optimize the value of each parameter.

The design parameters of the load cell can be determined during the FEM design process based on the allowable stress and strain associated with the applied load. The allowable stress at the maximum applied load should be roughly 50% of the produced stress of the chosen material to guarantee an acceptable linear response of the load cell and to avoid permanent strain in the load cell body that could cause inaccurate calibration [41]. In addition, the slot height should be less than the yield deflection to prevent the material’s fracture. In designing a strain gauge load cell with 6061 aluminum, it is desirable to design the load cell structure to generate a significant strain near the highest acceptable strain at the highest force to improve the overall sensitivity of the load cell [2]. The maximum allowable strain value of 1000 microstrain is suggested from the perspective of the yield stress and sensitivity of the load cell to ensure the linear response and performance of the strain gauge [46]. However, approximately 1 mV/V of sensitivity at the highest applied load was utilized in this article to offer allowable sensitivity and accuracy, as reported in [47].

## 4. Shape Optimization Consistent with the Finite Element Method (FEM)

The topology shape of the proposed load cell is optimized based on finite element analysis (FEA). Additionally, the stress and strain analysis of the load cell under the applied torque are considered significant for shape optimization. The shape optimization changes the physical parameters of the load cell’s structure (hole diameter and body thickness) within the allowable range of values, as shown in Table 1. ANSYS software (ANSYS Workbench 2022 R2, ANSYS Inc.) was employed in this study to design and evaluate the load cell’s shape consistent with the finite element method (FEM). In contrast, the load cell’s structural model was created by 3D CAD software (Autodesk Inventor professional 2022, Autodesk Inc.). 

The proposed load cell design utilizes 6061 aluminum alloy, the most popular aluminum grade available, because of its low-cost material, good strength, and machinability. The Young’s modulus, yield stress, and Poisson’s ratio of this aluminum alloy were 68.9 GPa, 276 MPa, and 0.33, respectively, which were applied to the ANSYS software.

**Figure 7:**
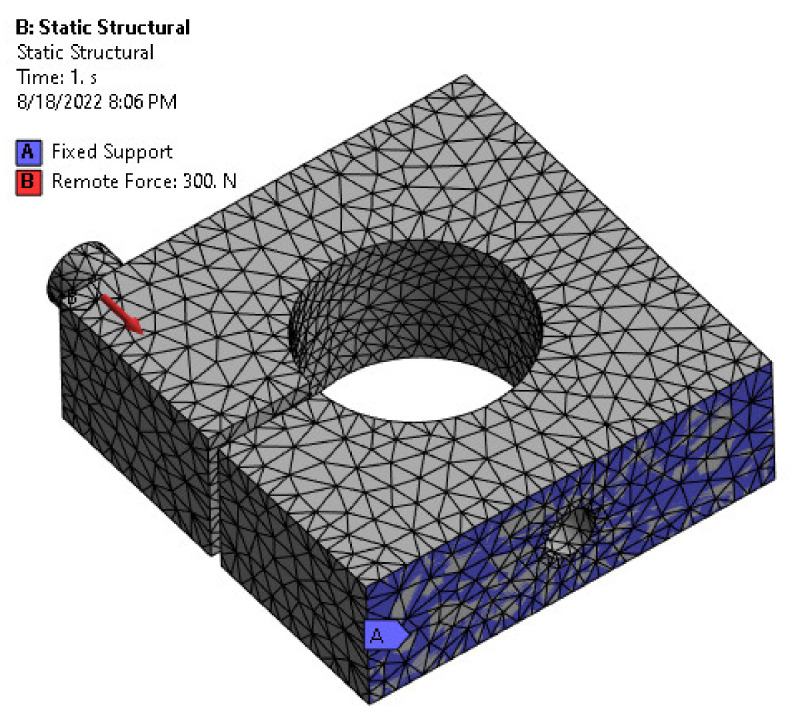
Meshed model along with boundary conditions and applied load.

In the analysis, two boundary conditions were defined based on the actual mounting operation of the load cell. As shown in Figure 7, since the load cell is fixed to the base with a bolt, a fixed support is applied to the bottom surface of the load cell, and the external force is applied to the load point at a distance of 5 mm from the nearest edge. The capacity of the load cell designed in this study is 300 N as the maximum applied force.

The load cell model is split into a tetrahedral mesh with a component size of 1 mm. The model’s element type is solid 187, with 14,110 nodes and 7747 finite elements. Element type solid 187 was chosen after investigating different types of elements and selecting the suitable number of elements based on the convergence test. Solid 187 element is a greater-order 3-D, 10-node component that exhibits a quadratic displacement performance, increasing the analysis’s accuracy [39]. The finite component mesh for the load cell model is shown in Figure 7.

**Figure 8:**
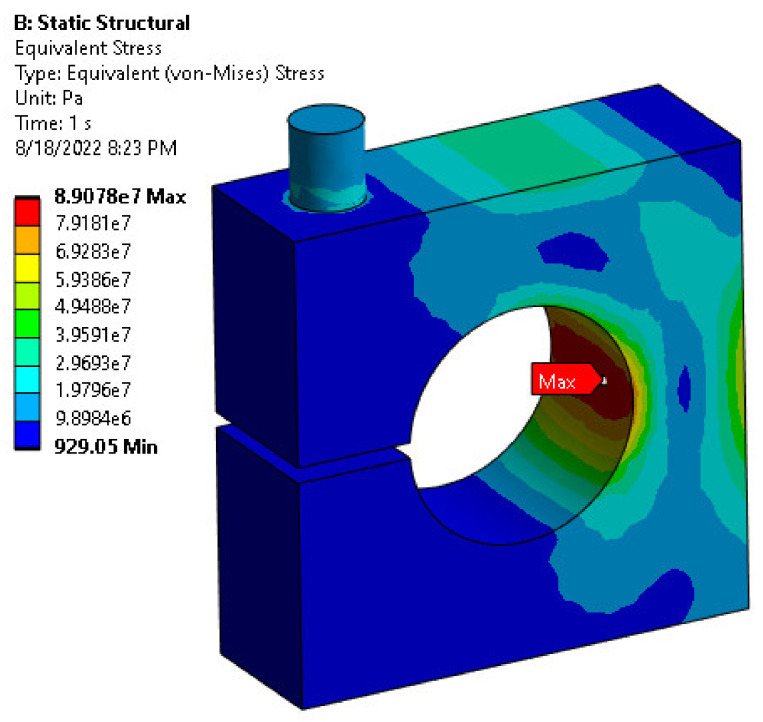
Von Mises stress FEM analysis.

**Figure 9:**
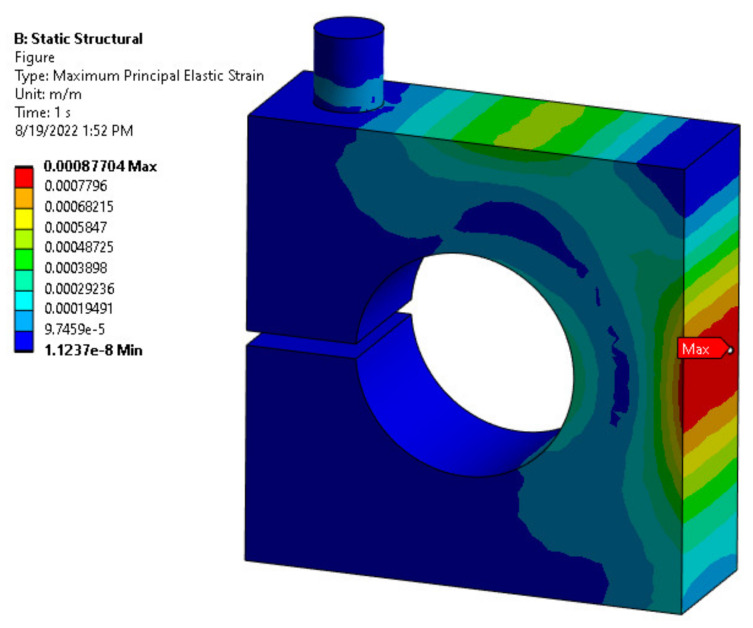
Maximum principal strain FEM analysis.

The stress–strain distribution with FEM analysis was carried out to ensure the maximum equivalent stress would be less than the material’s allowable strength under the applied load. At the same time, it was conducted to achieve the optimum values of the design parameters of the load cell while maintaining the required load cell characteristics. Thus, Figure 8 and Figure 9 show the association between the exerted force and generated von Mises stress and maximum principal strain within the allowable range of the hole diameter and body thickness values, respectively.

**Figure 10:**
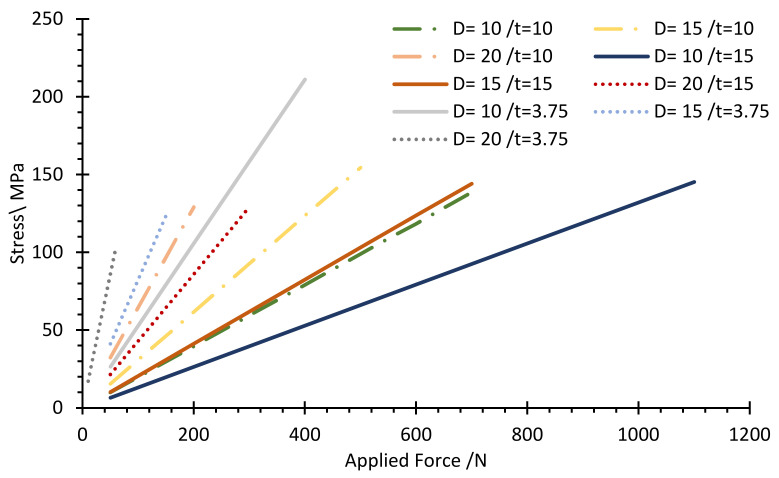
Von Mises stress versus applied force for the allowable range of parameters.

**Figure 11:**
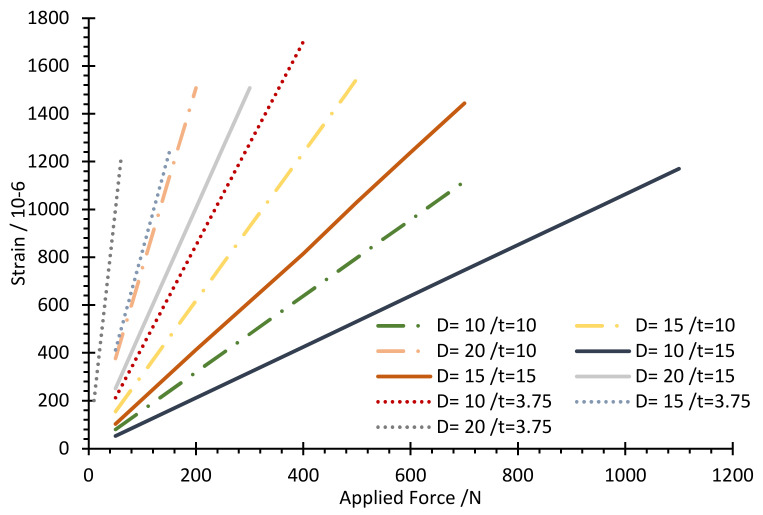
Maximum principal strain versus applied force for the allowable range of parameters.

**Figure 12:**
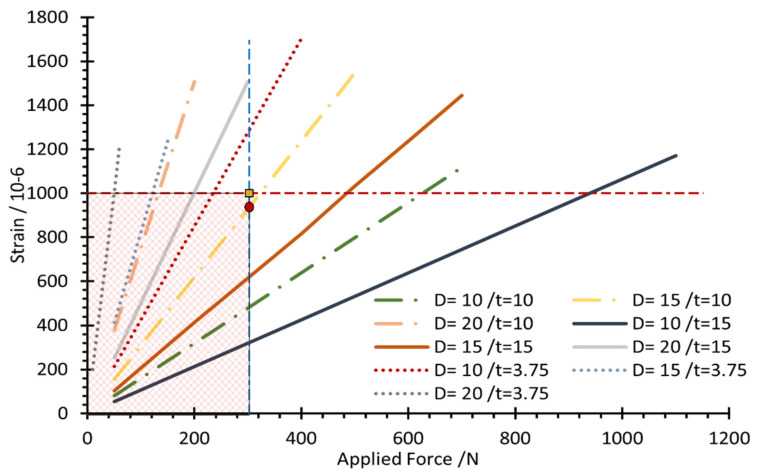
Limiting factors for the design parameters.

**Figure 13:**
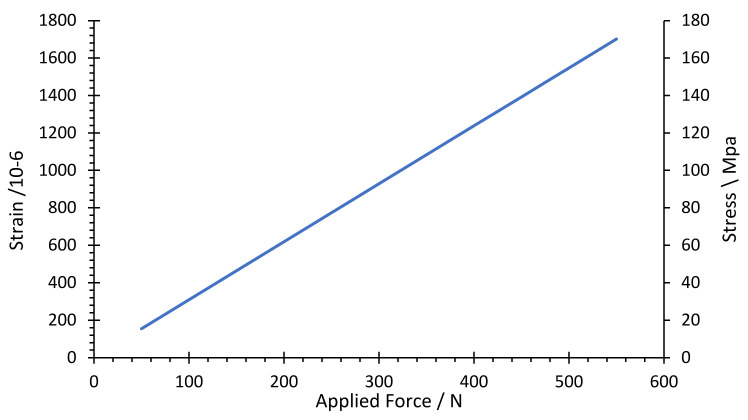
Stress and strain curve versus applied torque for the optimized parameters.

The limitation factors for choosing the optimal parameter value for the load cell are determined based on the allowable stress and strain, 138 MPa and 1000 microstrain, respectively. Figure 10 shows the allowable range of values determined based on the limiting factors. Therefore, the optimal values for the hole diameter (D) and body thickness (t) were selected as 15, and 10 mm, respectively, since it is the nearest line that could achieve the design values, as seen in Figure 11 and Figure 12, which show the relationship between the applied load and generated strain on the load cell for the optimized parameters through FEM analysis. Additionally, the FEM analysis of the model with these optimized parameters is displayed in Figure 8 and Figure 13.

## 5. Experimental Implementation

**Figure 14:**
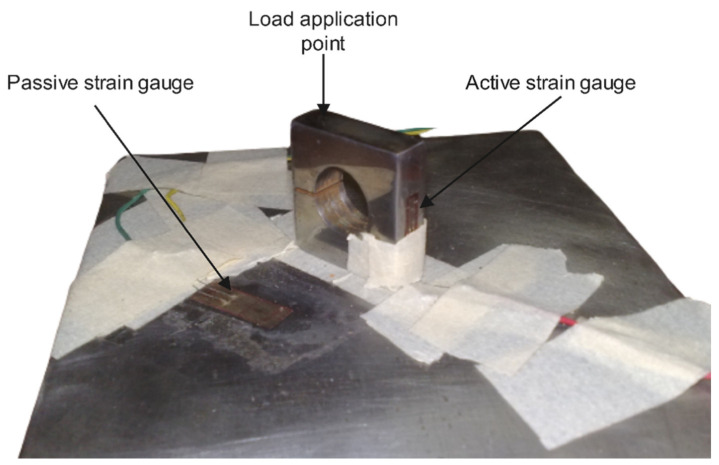
Load cell prototype.

A 10 mm metal plate (6061 aluminum) of the load cell prototype was manufactured by a CNC machine based on the optimized shape of the load cell. The load cell has a compact size (30 mm × 30 mm × 10 mm) and is lightweight (20 g). The single hole was drilled accurately at the center of the body to prevent any drifting during the machining. A small slot generated bending bridge strain on its opposite side. The manufactured load cell prototype is shown in Figure 14.

**Figure 15:**
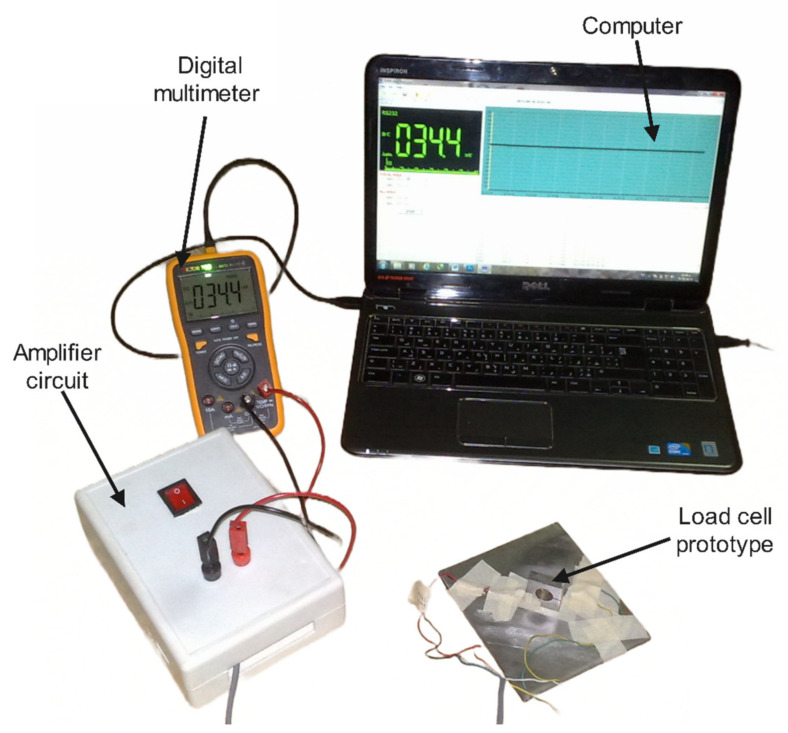
Experiment platform for load cell measurement.

The experimental platform was implemented for the force measurement of the load cell, as shown in Figure 15. The proposed work uses two strain gauges: the active gauge is placed on the side surface close to the bridge beam of the load cell body while the dummy gauge is mounted on the ground surface to minimize the adverse effects of temperature changes. The strain gauges are bonded with epoxy adhesive. The contact surface is lightly ground with sandpaper of 600 or finer grit before cleaning it with acetone. Then, the strain gauges are protected with a layer of epoxy.

**Figure 16:**
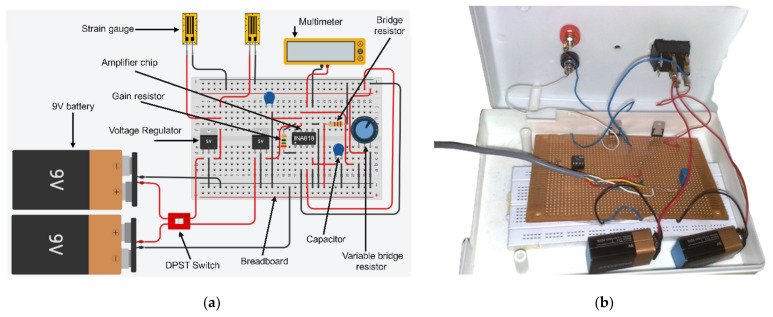
Implementation of the amplifier circuit: (**a**) Graphical description of the load cell circuit board; (**b**) Description of the actual amplifier circuit.

The actual implantation of the amplifier circuit is shown in Figure 16. The load cell is attached to a Wheatstone bridge instrument with an analog amplifier to increase the bridge’s signal level by a factor of 2001. This study utilized a linear strain gauge (CEFLA-3-23, Tokyo Measuring Instruments Laboratory Co., Tokyo, Japan) with a gauge length of 3 mm, a backing length of 6.9 mm, and a resistance of 120 Ω. The strain limit of this strain gauge is 1% (10,000 microstrain) and the gauge factor (GF) is 2.09 [45]. The other strain gauge specifications are special alloy foil for the grid, polyimide resin for the backing material, and self-temperature compensation for aluminum. The output of the amplifier circuit ($V_{o}$ and ground) is fixed to the instrument digital multimeter (Digital Multimeter VICTOR 70C). This multimeter interfaces with the computer by USB cable to display and record the data logging, processing, and analysis. The specifications of the amplifier circuit are illustrated in Table 2.

The load cell calibration was carried out in the experimental setting by employing known accredited mass loads at the load application spot to characterize the sensitivity and design of the load cell. These masses were verified based on the standard weight set specifications. These calibration tests are widely used in various load cell calibrations [39]. In the calibration experiment, two goals were pursued. First and foremost, measurement of the load cell’s sensitivity and linearity (also known as the calibration slope) was crucial. Secondly, the calibration should be carried out before any force measurement on the load cell to balance the bridge by adjusting the bridge’s output voltage through the variable resistor until the voltmeter reads zero volts. Primarily, the bridge’s output generated a non-zero initial offset voltage when no load was applied because of the slight variation in resistance among the bridge arms.

## 6. Results and Discussion

**Figure 17:**
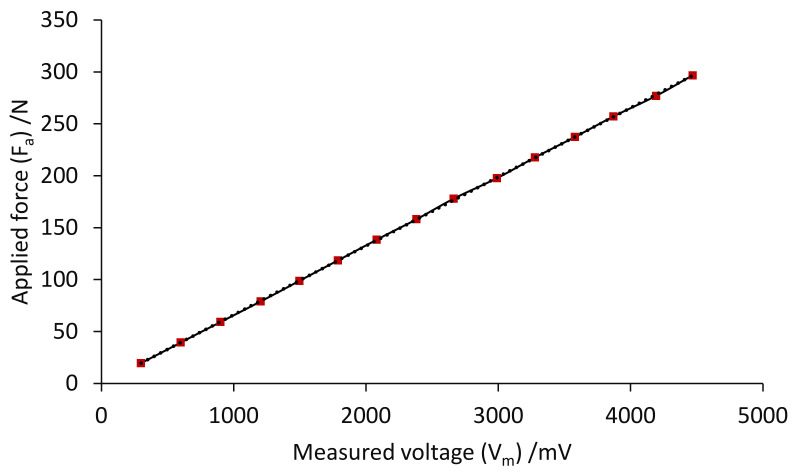
Calibration curve of applied force versus measured voltage.

In the calibration experiment test for load cell, the values of masses ranged from 2 to 30 kg, increasing in 2 kg increments. Each applied mass was repeated to eliminate random error and obtain accurate measurements. The average values of the three measured tests are shown in Figure 17. The calibration curve of the observed voltages related to the mass of the load set is shown in Figure 17. The load cell’s sensitivity was 15 mV/N, according to the slope of the line through the data points. The measured output voltage and corresponding sensitivity for each applied load are listed in Table 3; the highest sensitivity was taken at 446.8 µV/V at the maximum applied load. Ultimately, the governing equation of the applied load–output voltage relationship was obtained and derived according to the calibration experiment of the load cell, as follows:

(14) $$F_{a}=0.0664\;V_{m}-0.1981$$

where $F_{a}$ is the applied load (N) and $V_{m}\;$ is the measured voltage (mV).

The finding [39] that the calculated calibration constant coincides nicely with that obtained from calibrating the load cell in a standard testing machine supports the experimental results obtained by this work. The developed technique was validated through the use of several drop weight tests on the specimen form of the load cell. The proposed load cell has observed sensitivity that also matches the application’s specifications quite well and places it within the range of findings in [39].

**Figure 18:**
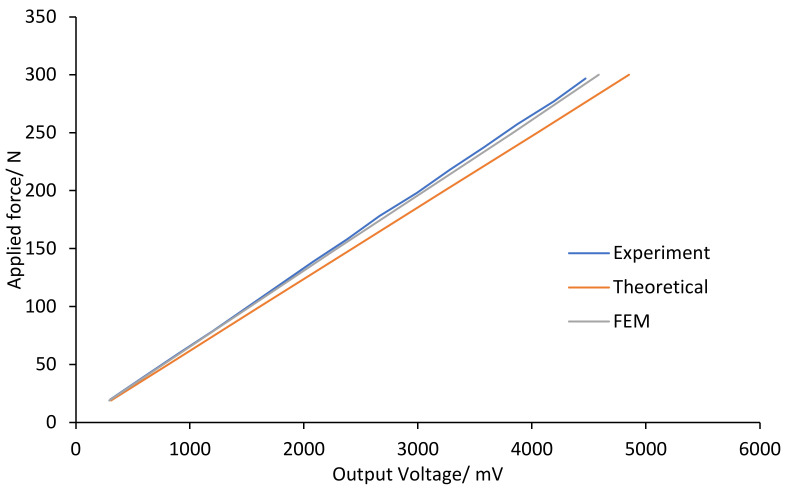
Output voltage versus applied load for the theoretical, FEM, and experiment.

On the other hand, the theoretical sensitivity of the load cell, 16 mV/N, can be obtained from the slope in Figure 18, which is consistent with the determined sensitivity of 15 mV/N. Figure 18 shows the theoretical, FEM, and experimental results of the correlation between the yield voltage and applied torque. The experiment and FEM results are almost identical, with around a 1.4% difference. Furthermore, the experiment and FEM results differed from the theoretical result by 6.7% and 5.4%, respectively. 

The results obtained in this study concord with those of [39], in which FEM integrated contact analysis, automatic meshing, and infinite elements were constructed on load cells. The simulation’s results strongly agree with the analytical solution, benchmark issues, and experimental findings. Predictions on the end-loading sensitivity of solid and load cells were carried out using the FEM software.

**Figure 19:**
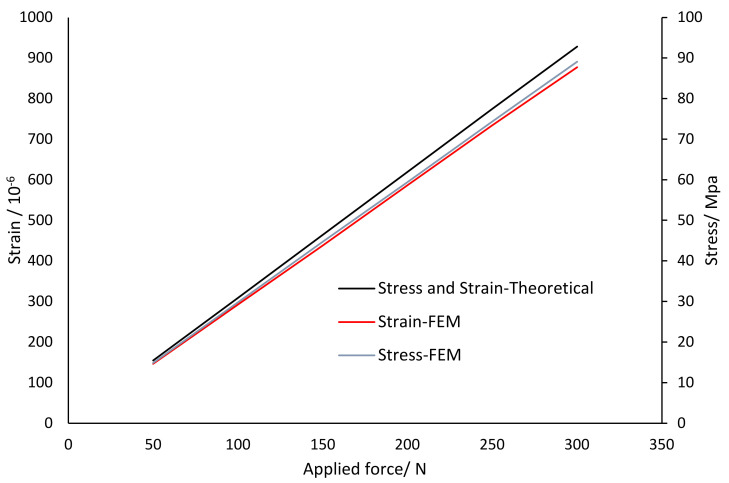
Stress and strain versus applied load for the theoretical and FEM.

The theoretical calculation was based on the mathematical model equations of the load cell and the simulation results, which were based on FEM. The stress and strain curve corresponding to the applied load is given in Figure 19 for the theoretical and simulation (FEM) results. In the strain and stress analysis, the simulation results are lower than the theoretical results by 5.4% and 3.7%, respectively. 

In summary, the experimental, simulation, and theoretical results confirmed each other, with an acceptable percentage of errors in the output voltage, stress, and strain results. There are reasons behind these differences and errors, such as miscalculations based on the properties of the used material, the experiment’s environmental circumstances, and the machining process’ residual stress. However, the designed and optimized load cell meets the design requirements and considerations with a specific load range. Using FEM, the strain gauge load cell was found to satisfy all criteria and to be sensitive only to axial loading, which supports the current optimization parameters and results.

## 7. Conclusions

The feasibility of designing and customizing the strain gauge load cell based on the specific design requirements and criteria is shown in this paper. The load cell was designed and optimized for measuring the axial force of laboratory test benches, which provides a 300 N load capacity, low-cost material, low weight, and size with highly accurate force measurement. The structured shape of the load cell was optimized based on stress–strain analysis of FEM to enhance the load cell characteristics by reducing the weight and boosting the sensitivity within the allowable load range. The strain and stress analysis showed that the FEM results are lower than the theoretical results by 5.4% and 3.7%, respectively.

The load cell prototype was fabricated and tested using a Wheatstone half-bridge circuit with two strain embedded strain gauges (120 Ω) and amplifier gain (2001 times). The load cell prototype was manufactured with a CNC machine from aluminum 6061 material. The calibration test results confirmed that the output voltage change corresponds to the applied load with a sensitivity of 15 mV/N and 446.8 µV/V at the tested measurement range up to 30 kg, slightly lower than the theoretical sensitivity of 16 mV/N. Additional experimental findings revealed that the load cell yield voltage differs slightly from the theoretical and simulation results by 6.7% and 1.4%, respectively. These results demonstrated that the load cell’s prototype meets the design requirement for axial force measuring for the bench test, which has a light weight of 20 g and a maximum axial force capacity of 300 N. The proposed half-bridge load cell configuration with the proper structural design offers excellent resolution and sensitivity while minimizing the adverse effects of temperature changes.

## Figures and Tables

**Table 1:** Design parameters.

Parameter	Minimum Limit	Maximum Limit
Body thickness [*t*]	3.75 [mm]	15 [mm]
Hole diameter [*D*]	10 [mm]	20 [mm]

**Table 2:** List of the parts for the amplifier circuit.

Item	Specification
Instrumentation Amplifier	INA818
Voltage Regulator, 5 V	LM78L05ACZFS-ND
DPST Switch	SW102-ND
bridge resistor (R)	120 Ω
Fixed gain resistor (RG)	25 Ω
Variable bridge resistor (Rv, potentiometer)	120 Ω
9 V battery strap connector	2243K-ND
1.0 Micro Farad capacitor	P2105-ND
Excitation voltage (Eex)	5 Volt
Amplifier Gain (G)	2001

**Table 3:** Calibration results of the load cell by experiment.

Load/kg (N)	Output Voltage/mV	Sensitivity µV/V
2 (19.7933)	300.30	30.00
4 (39.5866)	600.10	59.90
6 (59.3799)	900.80	90.00
8 (79.1732)	1205.00	120.40
10 (98.9665)	1507.00	149.60
12 (118.7598)	1809.00	178.80
14 (138.5531)	2101.00	207.90
16 (158.3464)	2413.00	238.20
18 (178.1397)	2705.00	266.30
20 (197.933)	3016.00	298.90
22 (217.7263)	3328.00	327.60
24 (237.5196)	3620.00	357.80
26 (257.3129)	3902.00	387.00
28 (277.1062)	4224.00	419.20
30 (296.8995)	4535.00	446.80

## Data Availability

Not applicable.
